# Preparation of Restricted Access Media-Molecularly Imprinted Polymers for the Detection of Chloramphenicol in Bovine Serum

**DOI:** 10.1155/2019/7930102

**Published:** 2019-12-12

**Authors:** Shanwen Zhao, Chanling Wei, Zhian Sun, Huachun Liu, Yanqiang Zhou, Xiaoxiao Wang, Jianmin Li, Bolin Gong

**Affiliations:** ^1^College of Chemistry and Chemical Engineering, North Minzu University, Yinchuan 750021, China; ^2^China Customs Ningxia Entry-Exit Inspection and Quarantine, Yinchuan 750000, China

## Abstract

Chloramphenicol- (CAP-) restricted access media-molecularly imprinted polymers (CAP-RAM-MIPs) were prepared by precipitation polymerization using CAP as a template molecule, 2-diethylaminoethyl methacrylate (DEAEM) as a functional monomer, ethylene glycol dimethyl acrylate (EDMA) as a crosslinking agent, glycidyl methacrylate (GMA) as an outer hydrophilic functional monomer, and acetonitrile as a pore former and solvent. The CAP-RAM-MIPs were successfully characterized by Fourier-transform infrared spectroscopy, scanning electron microscopy, and thermogravimetric analysis. The adsorption performance was investigated in detail using static, dynamic, and selective adsorption experiments. Adsorption equilibrium could be reached within 11 min. The CAP-RAM-MIPs had a high adsorption rate and good specific adsorption properties. Scatchard fitting curves indicated there were two binding sites for CAP-RAM-MIPs. Adsorption was Freundlich multilayer adsorption and consistent with the quasi-second kinetic model. Using CAP-RAM-MIPs for selective separation and enrichment CAP in bovine serum in combination with high-performance liquid chromatography (HPLC), CAP recovery ranged from 94.1 to 97.9% with relative standard deviations of 0.7–1.5%. This material has broad application prospects in enrichment and separation.

## 1. Introduction

Chloramphenicol (CAP) [1] is an antibiotic that can inhibit both Gram-negative and -positive bacteria and effectively control various animal diseases. It has significant antimicrobial and pharmacokinetic properties [2–4]. CAP residues in animals can be transmitted to humans through food and could predispose humans to various diseases. Due to its severe toxicity and side effects for the human immune and hematopoietic systems [5], CAP has been banned from animal breeding and aquatic products [6]. Currently, methods for detection of CAP residues include microbiological methods, chromatography [7–11], liquid-chromatography tandem mass spectrometry (LC-MS) [12, 13], and chromatography combined with immunoassay [14–16]. However, these methods have inevitable disadvantages [17]. For example, complex sample pretreatments such as liquid-liquid extraction are necessary before detection.

Molecular imprinting technology (MIT) is a new technology based on the antigen-antibody theory, which has foundations in biochemistry, polymer chemistry, and material science, as well as other disciplines [18–21]. Molecular imprinted polymers (MIPs) are functional materials with specific recognition abilities and have been widely used in the detection of CAP residues in various matrices such as milk, milk powder, serum, honey, shrimp, and urine [22]. These polymers have high-specificity recognition ability for template molecules [20, 23, 24]. However, traditional MIPs have some problems, such as slow mass transfer rate and low binding efficiency [25, 26]. Moreover, some components in biological samples, such as proteins, can be adsorbed onto the surface of MIPs through hydrophobic action and thereby affect the recognition ability of MIPs.

Therefore, hydrophilic functional groups can be modified on the surface of MIPs and a hydrophilic polymer layer formed can effectively reduce the adsorption of protein. Compared with general MIPs, restricted access media-molecularly imprinted polymers (RAM-MIPs) have abundant hydroxyl on the external surface, which can prevent blockage of the imprinting site and allow target small molecules to enter the imprinting site efficiently [27]. RAM-MIPs perfectly combine the advantages of RAM with MIPs, improve the selectivity of target small molecules, and expand the application scope of MIPs.

Victoria et al. [28] used CAP as a template molecule, 3-aminopropyltriethoxysilane (3-APTES) and triethoxyphenylsilane (TEPS) as the functional precursors, tetramethyl orthosilicate (TMOS) as the cross-linker, isopropanol as the solvent/porogen, and HCl as the sol-gel catalyst to synthesize sol-gel imprinted polymers with high CAP-specific selectivity performance, where the CAP-imprinting factor was very high. Ji et al. [29] first synthesized magnetic ferric oxide-modified methylallyl propyl trimethoxysilane and then prepared magnetic bisphenol A MIPs using microemulsion polymerization. The resulting polymer had a regular spherical structure and good magnetic susceptibility. A simple solid-phase microextraction method was established, which not only allowed convenient, economical, and efficient extraction but also overcame the problems associated with traditional solid-phase extraction column, such as column blockage and a large time requirement. New applications for these materials in many fields have attracted more and more attention.

In this study, the hydrophilic group (OH) on the surface of RAM-MIPs could effectively reduce the irreversible adsorption of protein macromolecules. CAP-RAM-MIPs had an increased adsorption capacity and selectivity. The prepared CAP-RAM-MIPs had good hydrophilicity, and it was combined with solid-phase extraction technology to pretreat bovine serum samples. Based on HPLC testing, satisfactory results have been achieved. This method facilitates simple, rapid, and economical detection CAP in bovine serum samples, which is of practical significance for detecting trace amounts CAP in biological samples to further ensure food safety, protect the environment, and promote human health.

## 2. Experimental

### 2.1. Materials and Reagents

Chloramphenicol (CAP), thiamphenicol (TAP), florfenicol (FP), disodium ethylenediamine tetraacetate (EDTA-2Na), diethylaminoethyl methacrylate (DEAME), and ethylene glycol dimethyl acrylate (EDMA) were purchased from Aladdin Reagent Co., Ltd. (Shanghai, China). Methanol and acetone were provided by Xi'an Chemicals Ltd. Azodiisobutyronitrile (AIBN), glacial acetic acid, acetonitrile, perchloric acid, and cuprous bromide (CuBr) were provided by Xi'an Chemicals Ltd. Bovine serum albumin (BSA) and bovine serum were purchased from Alfa Aesar Chemical Company.

We accurately weighed 20 mg of chloramphenicol and prepared a 2000 mg/L chloramphenicol standard solution. We dilute the solution step by step and then perform standard curve determination.

All other reagents are of analytical grade, and double distilled water was used throughout the experiment. Before use in HPLC analysis, the solution must be filtered through a 0.45 *μ*m nylon filter.

### 2.2. Apparatus and Conditions

Experiments were carried out using an H-2 digital constant temperature water bath (Changzhou Guohua Electric Co., Ltd.), SHZ-C water bath temperature oscillator (Shanghai Langgan Experimental Equipment Co., Ltd.), TGL-20M high-speed desktop centrifuge (Changsha Xiangyi Centrifuge Co., Ltd.), rotary evaporator (Shanghai Yarong Biochemical Instrument Factory), BS-224S electronic balance (Sartorius Scientific Instruments Co., Ltd.), JJ-1 precision factory electric blender (Shanghai Model Plant Manufacturing Co., Ltd.), KQ-3200E ultrasonic cleaner (Kunshan Ultrasonic Instruments Co., Ltd), JSM-7500F scanning electron microscope (JEOL Co. Japan), and TU-1810 UV-Vis spectrophotometer (Beijing General Analysis General Instrument Co., Ltd.). An LC-20AT high-performance liquid chromatography and Fourier-transform infrared spectrometer were purchased from the Shimadzu Corporation. The binary pump, variable wavelength UV detector, and a C18 (5 *μ*m particle size, 150 mm × 4.6 mm) analytical column were also purchased from the Shimadzu Corporation. The optimized mobile phase was methanol-water (4/2, v/v) at a flow rate of 0.6 mL/min. The detection wavelength was at 278 nm. The injection volume and column temperature were 20 *μ*L and 25°C, respectively.

### 2.3. Synthesis of CAP-RAM-MIPs

#### 2.3.1. Synthesis of Chloramphenicol-Restricted Molecularly Imprinted Polymers

CAP (0.2 mmol) was added to acetonitrile (40 mL) in a round-bottom flask, which was placed into an ultrasound machine to dissolve and evenly disperse the CAP. Functional monomer DEAEM (1.2 mmol) was added and prepolymerized at room temperature for 4 h. The crosslinking agent EDMA (7.2 mmol), the hydrophilic functional monomer GMA (1.2 mmol), and the initiator AIBN (25 mg) were added, the mixture was ultrasonicated, and then the round-bottom flask was filled with nitrogen for 30 min to remove oxygen. The mixture was incubated at 60°C to react for 24 h. After the reaction, the product was washed several times with methanol and acetonitrile and then dried in a vacuum oven.

#### 2.3.2. Chloramphenicol Molecular Imprinting Polymer Template Molecule Removal and Surface Epoxy Ring Opening

**Figure 1:**
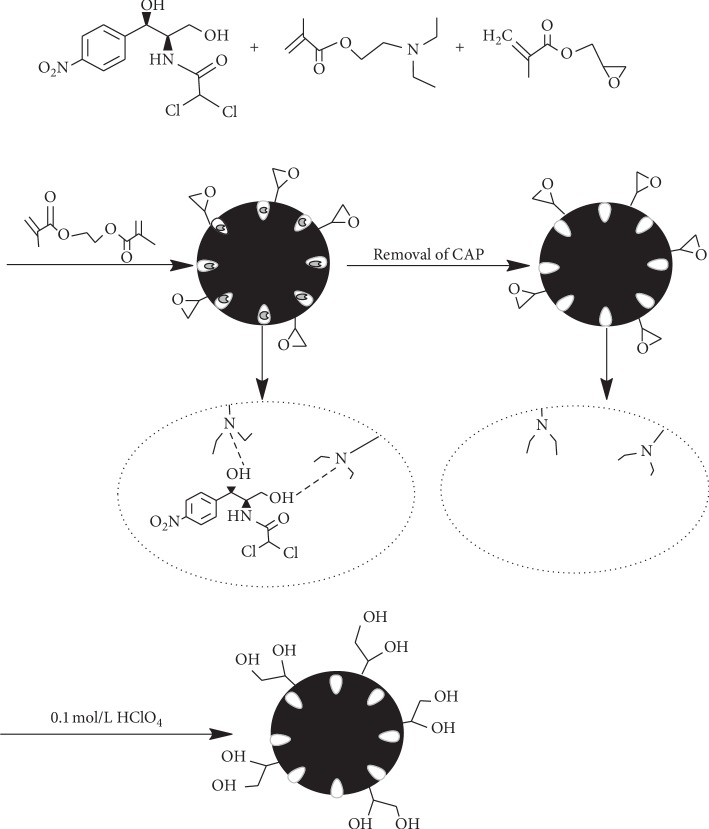
Synthesis process of CAP-RAM-MIPs.

The product was washed with methanol-acetic acid (9/1, v/v) as the eluting solvent in a soxhlet extraction. Soxhlet extraction washes away the template molecules and then washes the mixture to neutral with methanol. Finally, the CAP template was removed and the CAP-MIPs were obtained by drying in an oven. After the CAP-MIPs (500 mg) were added to a round-bottom flask, 50 mL of 10% perchloric acid solution was added and the mixture was stirred using magnetic stirrers at room temperature for 24 h. After the reaction, the product was washed with distilled water until neutral, washed with anhydrous ethanol, and then vacuum dried for 24 h at 50°C. The final product was CAP-RAM-MIPs. For comparison, CAP-restricted access media nonmolecularly imprinted polymers (CAP-RAM-NIPs) were prepared using the same procedure described above, except that no template molecule was added. The preparation process is shown in Figure 1.

### 2.4. Adsorption Experiment

#### 2.4.1. Isothermal and Kinetic Adsorption Test

CAP-RAM-MIPs (20 mg) were accurately weighed into a flask, and 0.4 to 2.0 mg/mL CAP standard solution was added and the mixtures shaken for 8 h at 25°C. The same procedure was used for CAP-RAM-NIPs. The liquid supernatant was measured by an ultraviolet visible spectrophotometer, and the adsorption amount was calculated according to the following formula:

(1) $$\begin{array}{c} Q=\frac{\left[\left(C_{0}-C_{\text{e}}\right)\;v\right]}{m}, \end{array}$$

where *Q* is the adsorption amount of polymer to CAP (mg/g), *C*_0_ is the initial concentration of CAP, *C*_e_ is the concentration at adsorption equilibrium (mg/mL), *v* is the solution volume (mL), and *m* is the mass of CAP-RAM-MIPs (g).

The equilibrium adsorption capacity *Q*_e_ of the CAP-RAM-MIPs was calculated according to the equilibrium concentration *C*_e_ as measured by static adsorption experiments. The adsorption isotherms were fitted using the Langmuir (2) and Freundlich (3) isotherm adsorption equations.

  Langmuir equation:

(2) $$\begin{array}{c} \frac{C_{\text{e}}}{Q_{\text{e}}}=\left(\frac{C_{\text{e}}}{Q_{\text{m}}}\right)+{\left(K_{\text{l}}Q_{\text{m}}\right)}^{-1}, \end{array}$$

  Freundlich equation:

(3) $$\begin{array}{c} \ln\;Q_{\text{e}}=n^{-1}\ln\;C_{\text{e}}+\ln\;K_{\text{F}}, \end{array}$$

where *C*_e_ (mg/mL) is the equilibrium concentration of CAP, *Q*_m_ is the maximum theoretical adsorption number of CAP-RAM-MIPs (mg/g), *K*_l_ is the Langmuir adsorption equilibrium constant (L/mg), *K*_F_ is the adsorption capacity (mg/g) of CAP-RAM-MIPs, *n* is the affinity of CAP-RAM-MIPs for CAP, and *Q*_e_ is the equilibrium adsorption amount (mg/g) of CAP-RAM-MIPs.

The Scatchard equation is often used to study the adsorption behavior of imprinted polymers. The adsorption data were further processed according to Scatchard equation to estimate the binding properties of CAP-RAM-MIPs and CAP-RAM-NIPs. The Scatchard equation was as follows:

(4) $$\begin{array}{c} \frac{Q}{C_{\text{e}}}=\frac{\left(Q_{\max}-Q\right)}{K_{\text{D}}}, \end{array}$$

where *Q*_max_ (mg/g) is the maximum apparent adsorption amount and *K*_D_ (mg/mL) is the equilibrium dissociation constant.

As an SPE adsorbent, the binding kinetics of imprinted polymers are particularly important. Therefore, we investigated the relationship between the adsorption amount *Q* of CAP-RAM-MIPs and CAP-RAM-NIPs and time (*t*). To examine the adsorption rate of the CAP-RAM-MIPs, the polymers (20 mg) were dispersed in 10 mL of CAP solution (1.6 mg/mL) and then the mixture was shaken at 25°C. The concentration after adsorption was measured every minute, and adsorption amount by CAP-RAM-MIPs at different time points was calculated according to formula (1).

Quasi-first-order and quasi-second-order models were used to describe the adsorption kinetics of CAP-RAM-MIPs for CAP. The two models were as follows:

(5) $$\begin{array}{c} \ln\left(Q_{\text{e}}-Q_{\text{t}}\right)=\ln\;Q_{\text{e}}-K_{1}t,\\ \frac{t}{Q_{\text{t}}}={\left(K_{2}Q_{e}^{2}\right)}^{-1}+\frac{t}{Q_{\text{e}}}. \end{array}$$

where *Q*_e_ (mg/g) is the equilibrium adsorption capacity, *Q*_t_ (mg/g) is the adsorption capacity at *t* (min), and *K*_1_ (min^−1^) and *K*_2_ (g mg^−1^·min^−1^) are the quasi-first and quasi-second-order rate constants, respectively.

#### 2.4.2. Selectivity Studies

Selective adsorption of CAP-RAM-MIPs was assessed using structural analogs TAP and FP to study the specific recognition performance of CAP-RAM-MIPs.

CAP-RAM-MIPs (20 mg) were accurately weighed to add in 3 groups of bottles. Methanol solutions (10 mL) of 1.5 mg/mL CAP, TAP, or FP were added in three group bottles. The mixtures were oscillated for 8 h at room temperature, and then adsorption amount was calculated according to formula (1), CAP-RAM-NIPs were measured using the same method.

Specific adsorption is one of the characteristics of molecularly imprinted polymers. The specificity of CAP-RAM-MIPs is usually analyzed based on partition, selectivity, and relative selectivity coefficients:

(6) $$\begin{array}{c} K_{\text{d}}=\frac{Q}{C_{\text{e}}}, \end{array}$$

(7) $$\begin{array}{c} K=\frac{K_{\text{d}1}}{K_{\text{d}2}}, \end{array}$$

(8) $$\begin{array}{c} K^{\prime }=\frac{K_{\text{MIP}}}{K_{\text{NIP}}}, \end{array}$$

where *K*_d_ is the distribution coefficient, *K* is the selective coefficient of CAP-RAM-MIPs and CAP-RAM-NIPs for CAP, *K*_d1_ is the partition coefficient of CAP, *K*_d2_ is the partition coefficient of competitors, and *K*′ is the relative selectivity coefficient of CAP-RAM-MIPs and CAP-RAM-NIPs.

### 2.5. Recycling Performance of RAM-MIPs

To investigate the reusability of CAP-RAM-MIPs, CAP-RAM-MIPs-SPE cartridges were prepared. Several repeated adsorption and elution tests were carried out to determine the reuse performance.

### 2.6. Evaluation of Protein Exclusion Efficiency

The packed CAP-RAM-MIPs-SPE columns were activated with methanol and water in turn and injected with 1 mg/mL BSA solutions, and the effluent liquids were collected. The BSA concentration was measured at 280 nm, and the binding ability of CAP-RAM-MIPs and CAP-MIPs for BSA was calculated.

### 2.7. Effect of Temperature on Adsorption Capacity

CAP-RAM-MIPs (20 mg) were weighed in a flask, and 10 mL CAP solution (1.5 mg/mL) was then added. The mixture was shaken at either 15, 25, or 35°C for 2 h. The amount of adsorption was calculated using formula (1).

### 2.8. Actual Sample Determination

The concentration of 1 mg/L∼20 mg/L chloramphenicol standard solution was prepared with methanol, and the standard curve was determined by HPLC. 0.2 mL serum was mixed with 0.8 mL water to prepare the bovine serum standard solution with chloramphenicol concentrations of 2 mg/L, 10 mg/L, and 20 mg/L. Sample solutions with different scalers were added to CAP-RAM-MIPs-SPE columns previously activated with 3 mL methanol and 3 mL water and then leached with 1 mL water. The elution was performed with 2 mL methanol-glacial acetic acid (9/1, v/v) solution. The elution was evaporated to dryness on a rotary evaporator and redissolved with 1 mL of mobile phase, and the solution was detected by HPLC.

## 3. Results and Discussion

### 3.1. Characterization of CAP-RAM-MIPs

**Figure 2:**
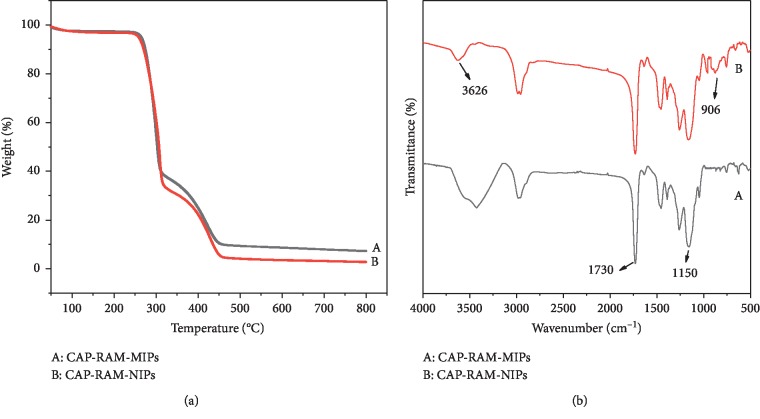
(a) Thermogravimetric and (b) FT-IR spectrum curves of (A) CAP-RAM-MIPs and (B) CAP-RAM-NIPs.

Figure 2(a) shows the weight loss of the two polymers. The weight loss was about 2.5% in the range of 25–110°C with the main component lost being water. The rapid weight loss of polymers at 300–500°C can be attributed to the decomposition of organic matter. The results show that CAP-RAM-MIPs have good thermal stability.  Figure 2(b) shows the infrared spectra of CAP-RAM-MIPs and CAP-MIPs, where A and B are the CAP-RAM-MIPs and CAP-MIPs, respectively. The infrared data are a good indication of whether the material has been synthesized successfully or not. The peak at 3626 cm^−1^ is the stretching vibration peak of -OH formed by epoxy ring opening with the peak strength enhanced in the graph, the one near the 906 cm^−1^ is the vibration peak formed by the epoxy group, and the intensity is obviously weakened after the opening of the ring, indicating opening of the epoxy ring was successful. The peak at 1730 cm^−1^ is the vibration peak of –C=O. The peaks at 1150 cm^−1^ are the asymmetric stretching vibration peaks of C-O-C and 4000–2500 cm^−1^ are the hydrogen bond regions of O-H, N-H, and C-H, where strong hydrogen bonds can be seen in the infrared spectrum. In summary, infrared spectroscopy confirmed the existence of large number of hydroxyl groups on the polymer surface that endow the polymer surface with hydrophilic properties.

**Figure 3:**
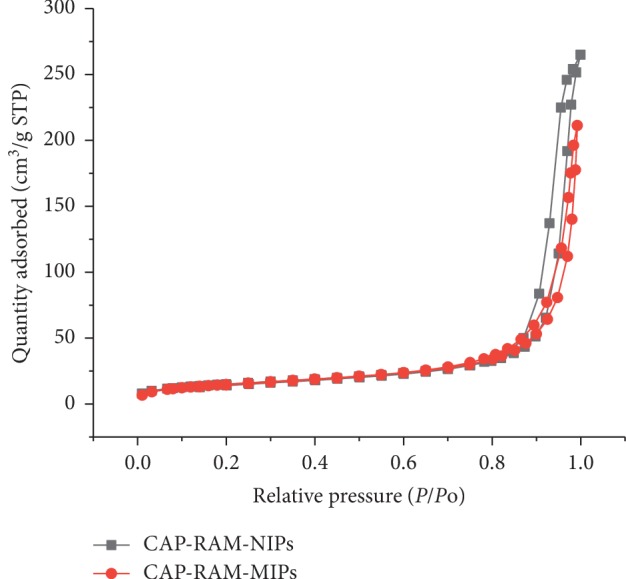
N_2_ adsorption of CAP-RAM-MIPs and CAP-RAM-NIPs.

Figure 3 presents N_2_ adsorption of CAP-RAM-MIPs and CAP-RAM-NIPs. The specific surface area of the CAP-RAM-MIPs and CAP-RAM-NIPs was 53.475 m^2^/g and 53.243 m^2^/g, respectively. There was no significant difference in the specific surface area between the two. However, the pore size distribution is significantly different. The CAP-RAM-MIPs have a narrow pore size distribution and a wide CAP-RAM-NIP pore size distribution. This is because the template molecules are added during the preparation of the CAP-RAM-MIPs, and the pore size is relatively regular, while the CAP-RAM-NIPs are prepared without template molecules, the pores are randomly formed, and the shape is irregular.

### 3.2. Isothermal and Kinetic Adsorption

**Figure 4:**
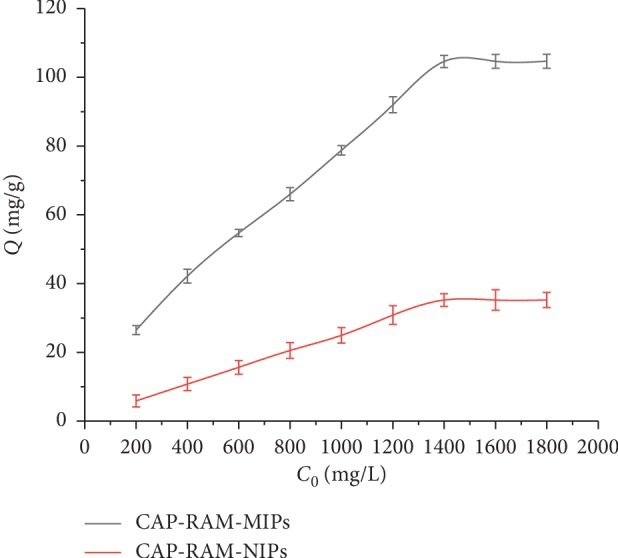
Isotherms adsorption amount of CAP by CAP-RAM-MIPs and CAP-RAM-NIPs.

From Figure 4, it can be seen the adsorption capacity of CAP-RAM-MIPs increased as the CAP concentration increased at 25°C, with saturation being reached around 1.0 mg/mL and a maximum adsorption capacity of polymer for CAP was 104.67 mg/g. There was more significant adsorption by the CAP-RAM-MIPs than CAP-RAM-NIPs at a given concentration. CAP-RAM-MIPs with spaces matching CAP were prepared, where the loci imprinted for CAP had “memory” function that allowed specific recognition of CAP. The CAP-RAM-NIPs did not have such molecularly imprinted loci, resulting in the adsorption capacity being low. This finding indicated that CAP-RAM-MIPs have strong affinity for CAP, which is due to the concept that the adsorption of CAP by RAM-MIPs is primarily based on specific adsorption, generating a strong adsorption capacity, while the CAP-RAM-NIPs only exhibited general physical adsorption to the CAP. The specific surface area of the two is not that different, but the adsorption amount is different. Thus, the adsorption difference between RAM-NIPs and RAM-MIPs was caused by the imprinting not by the surface area. Therefore, the difference in the adsorption ability of the two polymers is due to the different specificities of the imprinted sites.

**Figure 5:**
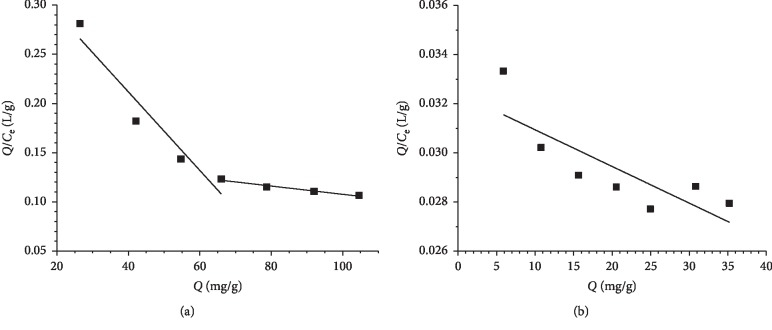
Scatchard plots of (a) CAP-RAM-MIPs and (b) CAP-RAM-NIPs.

According to the CAP-RAM-MIP and CAP-RAM-NIP isothermal adsorption data, *Q* was plotted with *Q/C*_e_ (Figure 5). The isothermal adsorption data of CAP-RAM-MIPs were calculated by Scatchard equation, and the *K*_d_ and *Q*_max_ could be calculated from the slope and intercept of the line plotted in *Q*/*C*_e_ versus *Q*. Comparing scatchard analysis curve of CAP-RAM-MIPs and CAP-RAM-NIPs, the curves of CAP-RAM-MIPs consist of two straight lines with different slopes. It showed that the adsorption for CAP by CAP-RAM-MIPs was not completely equivalent. There were two different binding sites. One was the binding site with strong affinity, and the other was the binding site with weak affinity. The dissociation constant *K*_d_ was 6711.4 g/L. The theoretical maximum adsorption capacity for CAP was 217.65 mg/g.

The Freundlich and Langmuir equations can be used to describe adsorption within a certain experimental range. The fitting results show the adsorption process was more consistent with Freundlich adsorption. Langmuir and Freundlich adsorption isotherm parameters are shown in Table 1.

**Figure 6:**
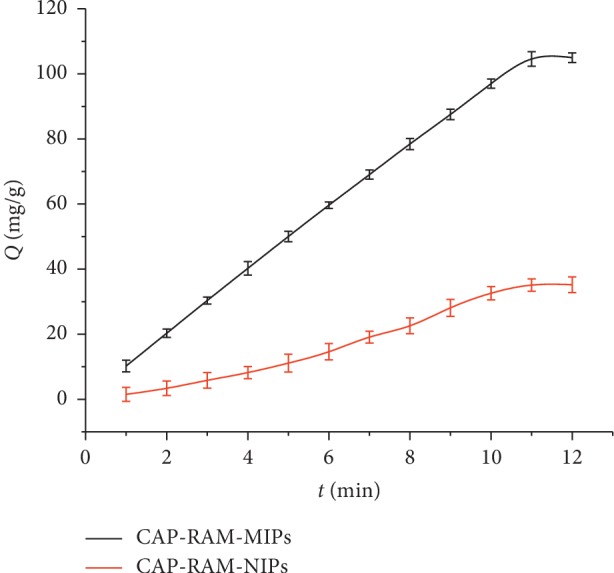
Dynamic adsorption curves of CAP-RAM-MIPs and CAP-RAM-NIPs.

It can be seen from Figure 6 that CAP-RAM-MIPs had very fast CAP adsorption rate and reached equilibrium within 11 min. At the same time, it was clear that the adsorption of the CAP-RAM-MIPs was higher than that of CAP-RAM-NIPs due to the specific binding sites on the surface for CAP. This is because the recognition sites produced by the surface imprinting of CAP-RAM-MIPs are distributed on the polymer surface and therefore have strong accessibility and low mass transfer resistance, allowing for quick attainment of adsorption equilibrium. These qualities provide incomparable advantages in sample pretreatment for an SPE adsorbent.

The fitting kinetic parameters are shown in Table 2, and the reaction series was determined according to the difference of the *Q*_e_ and *R*^2^. It can be seen from the table that CAP-RAM-MIP adsorption is more consistent with the quasi-second-order equation. The average theoretical adsorption quantity (*Q*_e_,_cal_) is close to the actual measured value (*Q*_e_,_exp_), and *R*^2^ is approximately equal to 0.999.

### 3.3. Adsorption Selectivity

**Figure 7:**
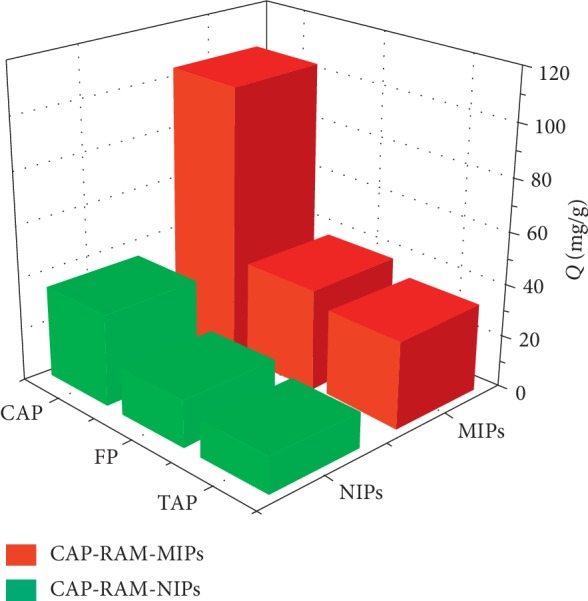
Selective adsorption of CAP, TAP, and FP by CAP-RAM-MIPs and CAP-RAM-NIPs.

Specific adsorption is a characteristic of CAP-RAM-MIPs. The selective adsorption experimental results are shown in Figure 7. The specificity of polymers is usually investigated using distribution, selectivity, and relative selectivity coefficients using formulas (6)–(8).

As shown in Table 3, the allocation and selectivity coefficients (*K*) of CAP-RAM-MIPs and CAP-RAM-NIPs for CAP, TAP, and FP were calculated. The *K*_D_ of CAP was 106.5, indicating the prepared CAP-RAM-MIPs had good specific recognition. We found that the adsorption capacity of RAM-MIPs on the CAP template molecule was the greatest and that of TAP was the smallest. We believe this finding was due to the presence of complementary cavities on the surface of the imprinted polymer, which had a preferential “response” to CAP, such that it would selectively absorb. The adsorption capacity of RAM-NIPs to the three drug molecules was similar, because the distribution of functional groups in nonimprinted polymers is arbitrary, the adsorption process is general physical adsorption, and there is no selectivity.

**Figure 8:**
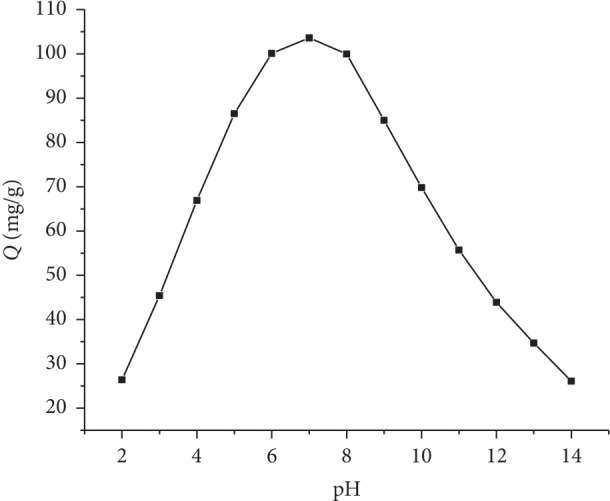
The effect of pH on the amount of adsorption.

We studied the relationship between pH and the amount of adsorption. As shown in Figure 8, the higher and lower pH have a great influence on the amount of adsorption. pH is about 7 or so, and the amount of adsorption reaches the maximum.

### 3.4. Reuse Performance of MIPs-SPE

The reusability is also an important criterion to evaluate the performance of CAP-RAM-MIPs. In order to investigate the reusability of CAP-RAM-MIPs-SPE, the experiment was repeated using the same column several times.

**Figure 9:**
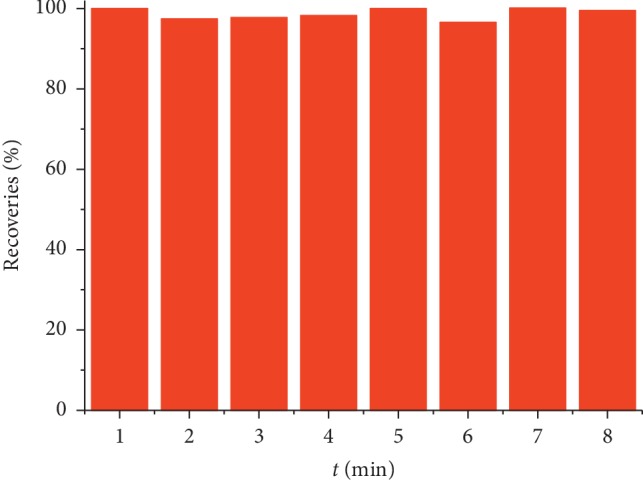
CAP-RAM-MIPs-SPE performance for repeated use.

As can be seen from Figure 9, after using the CAP-RAM-MIPs-SPE repeatedly 8 times, the recovery rate was above 96%. Based on this, CAP-RAM-MIPs-SPE is a good reusable SPE adsorbent, which can notably reduce the experimental cost. As an SPE filler, the CAP-RAM-MIPs have great advantages in the pretreatment of biological samples.

### 3.5. Exclusion Analysis

BSA is a polypeptide molecule with a high-molecular weight. BSA has previously been used as a protein model for in vitro studies and therefore was selected as the model for removal of protein macromolecules in this study. As can be seen from Table 4, the adsorption rate of BSA by CAP-MIPs was as high as 72.4%, while that of the CAP-RAM-MIPs was 0.9%, demonstrating the surface of the CAP-RAM-MIPs was rich in ethylene glycol groups and capable of blocking binding of macromolecular proteins.

**Figure 10:**
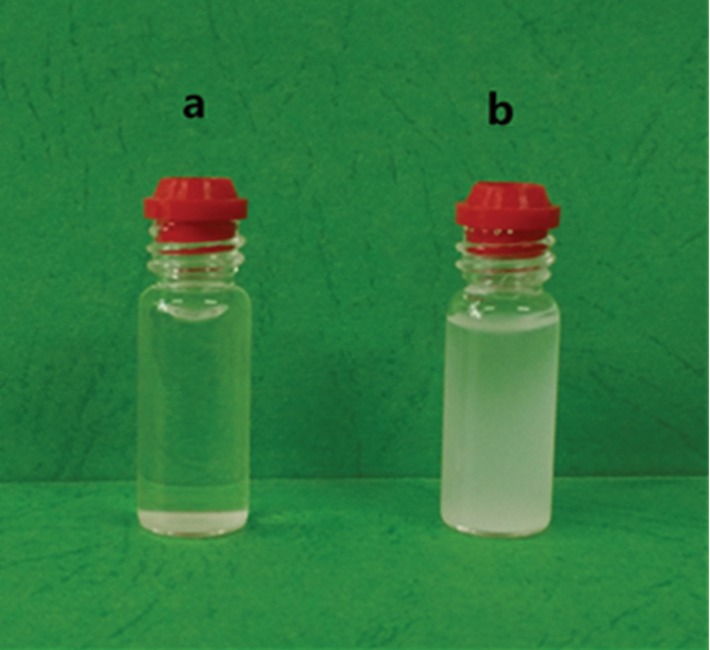
Photograph of microsphere dispersion in pure water after settling for 2 h: (a) CAP-MIPs and (b) CAP-RAM-MIPs.

After the preliminarily synthesized CAP-RAM-MIPs were treated with 10% perchloric acid, hydrophilicity was assessed based on the settlement of polymer in water. Ultrasonic dispersion of CAP-RAM-MIPs and CAP-MIPs was uniform, and the suspension state after 2 h of static suspension is shown in Figure 10(a) for CAP-MIPs and Figure 10(b) for CAP-RAM-MIPs. The CAP-MIPs settled faster in water, while the CAP-RAM-MIPs more easily and evenly dispersed in water, and thus the latter had better hydrophilicity. This further verifies the successful introduction of abundant hydrophilic groups onto the polymer surface. The reason for this is the presence of a large number of hydroxyl groups on the surface of the polymer after ring opening, which can create strong hydrophilicity and play an exclusive role in protein adsorption.

### 3.6. Effect of Temperature on Adsorption Capacity

**Figure 11:**
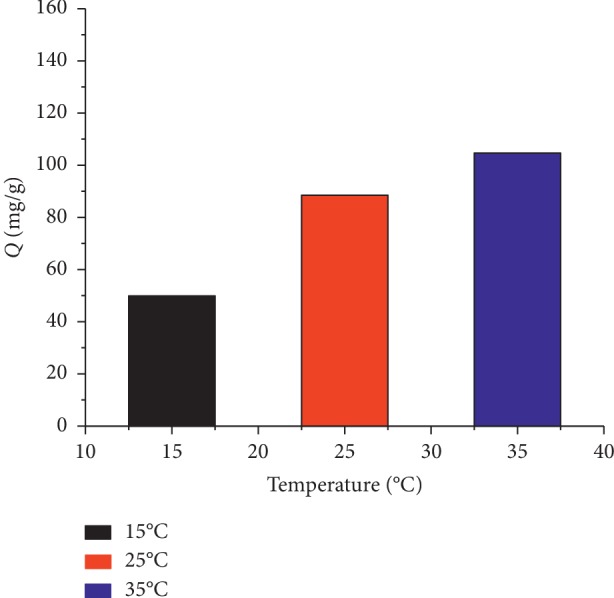
The relationship between adsorption amount and temperature.

Temperature is an important factor affecting the adsorption quantity. Therefore, the CAP adsorption capacity of the CAP-RAM-MIPs was investigated at 15, 25, and 35°C. As shown in Figure 11, the polymer adsorption quantity gradually increased as the temperature increased, suggesting the adsorption process is endothermic.

### 3.7. Analysis of Actual Samples

**Figure 12:**
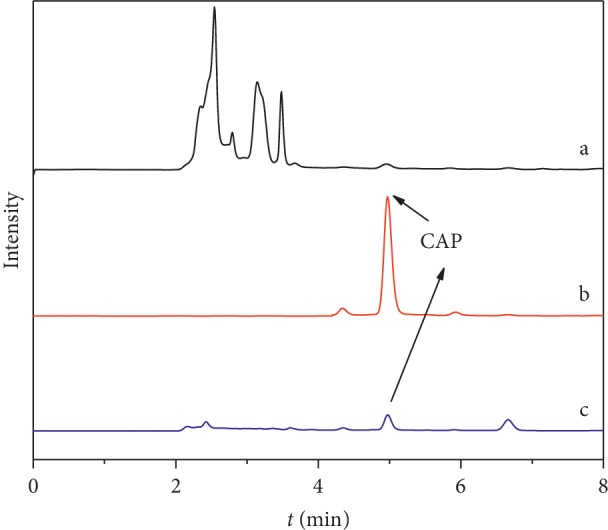
HPLC chromatograms of bovine serum samples. (a) Blank sample; (b) spiked sample solution extracted with the CAP-RAM-MIPs; (c) spiked sample solution extracted with the CAP-RAM-NIPs.

Based on the CAP-RAM-MIP adsorption experiment, the prepared material could adsorb large amounts of target molecule and undergo fast mass transfer. Using this material in combination with SPE technology, CAP in bovine serum samples was detected and analyzed by HPLC. The results are shown in Figure 12.

Under the same conditions, bovine serum samples containing 2, 10, and 20 mg/L CAP were treated with a CAP-RAM-MIPs-SPE column and CAP was detected by HPLC. The recoveries from the bovine serum samples and associated relative standard deviations are shown in Table 5. The detection limit was 1.2 *μ*g/L with a relative standard deviation of 0.7–1.5%, and the recoveries were 94.1–97.9%. This result indicated that the CAP-RAM-MIP-SPE could be used for the separation and enrichment of CAP in bovine serum. We compared the RAM-MIPs method with other reported methods for the determination of CAP. The results of this comparison are shown in Table 6 [30–33]. The data showed that the method had better sensitivity, higher adsorption speed, larger adsorption capacity, smaller standard deviation, and higher recovery than previously reported methods. Therefore, we believe that CAP-RAM-MIPs have thus been proven to be an efficient adsorbent.

## 4. Conclusions

In this study, CAP-RAM-MIPs were prepared by precipitation polymerization. The CAP-RAM-MIP structure and morphology were characterized by Fourier-transform infrared spectroscopy, thermogravimetry, and scanning electron microscopy. The adsorption properties of the CAP-RAM-MIP were investigated in depth through static, kinetic, and selective adsorption experiments and thermodynamics. The adsorption of CAP onto CAP-RAM-MIPs can be well described using the Freundlich isothermal adsorption equation. Selective experiments revealed the prepared CAP-RAM-MIPs had good recognition specificity.

BSA was selected as the macromolecular model, and SPE columns were used to confirm the CAP-RAM-MIP surface had abundant ethylene glycol groups and the ability to block macromolecular proteins. The CAP recovery by CAP-RAM-MIP columns was still higher than 96% after being reused 8 times, demonstrating good reusability. A combination of polymers and HPLC was used to separate and detect CAP in bovine serum. The method established in this study simplifies the pretreatment of samples, improves the selectivity of target molecules, and greatly reduces the experimental expense, demonstrating it has broad prospects in practical applications.

## Figures and Tables

**Table 1:** Adsorption isotherm constants for Langmuir and Freundlich equation.

Langmuir isotherm			Freundlich isotherm		
*Q*_m_ (mg/g)	*K*_L_ (L/mg)	*R* ^2^	*n*	*K*_F_ (g/L)	*R* ^2^
153.6	0.001688	0.9556	1.817	2.145	0.9872
*C*_e_/*Q*_e_ = 0.006510*C*_*e*_ + 3.855			ln *Q*_e_ = 0.5503*nC*_e_ + 0.7632		

**Table 2:** The results of kinetics analysis of CAP-RAM-MIPs.

Model	*K*	*Q*_e_,_cal_ (mg/g)	*Q*_e,exp_ (mmol/g)	*R* ^2^
Pseudo-first-order kinetic model	0.2488	158.25	104.7	0.8974
Pseudo-second-order kinetic model	0.3400	165.4	104.7	0.9933

**Table 3:** The distribution coefficients, selectivity factors, and relative selectivity coefficients of CAP-RAM-MIPs and CAP-RAM-NIPs.

Molecules	*C*_o_ (mg/mL)	*Q*_MIP_ (mg/g)	*Q*_NIP_ (mg/g)	*K*_D MIPs_	*K*_D NIPs_	*K*	IF
CAP	1.4	104.6	35.20	106.5	27.90	3.817	2.97
TAP	1.4	33.96	14.14	26.80	10.53	2.553	—
FP	1.4	38.90	18.10	31.26	13.63	2.293	—

**Table 4:** Bovine serum albumin adsorption rate of CAP-RAM-MIPs.

Materials	BSA concentration (mg/mL)	Absorbance		BSA binding rate (%)
		Before SPE	After SPE	
CAP-RAM-MIPs	1.0	0.584	0.579	0.9
CAP-MIPs	1.0	0.584	0.161	72.4

**Table 5:** Spiked recoveries of bovine serum samples.

Spiked concentration (mg/mL)	Rate of recoveries, *R* (%)			Average recoveries, $\bar{R}$ (%)	RSD (%)
	1	2	3		
2	94.5	92.6	95.1	94.1	1.47
10	95.9	96.6	97.3	96.6	0.7
20	96.4	99.1	98.2	97.9	1.37

**Table 6:** Comparison of CAP-MIPs for detection in bovine serum samples with existing reports.

Preparing methods	Analyte	Limit of detection (*μ*g/L)	RSD (%)	Recoveries (%)	Reference
Surface imprinting	CAP	5	<4.93	81–90	[30]
Surface imprinting	TC, CAP	—	—	72.9–83.6	[31]
Suspension polymerization	CAP	10	1.21–2.6	95.3–106.8	[32]
Surface-initiated atom transfer radical polymerization	OFL	0.2	2.47–3.3	95.6	[33]
Precipitation polymerization	CAP	1.2	0.7–1.5	94.1–97.9	This work

## Data Availability

The data used to support the findings of this study are available from the corresponding author upon request.
